# Ephaptic communication in the vertebrate retina

**DOI:** 10.3389/fnhum.2013.00612

**Published:** 2013-09-23

**Authors:** Rozan Vroman, Lauw J. Klaassen, Maarten Kamermans

**Affiliations:** ^1^Retinal Signal Processing, Netherlands Institute for NeuroscienceAmsterdam, Netherlands; ^2^Neurogenetics, University of Amsterdam - Academic Medical CenterAmsterdam, Netherlands

**Keywords:** ephaptic communication, vertebrate retina, cones, horizontal cells, inhibition

## Abstract

In the vertebrate retina, cones project to the horizontal cells (HCs) and bipolar cells (BCs). The communication between cones and HCs uses both chemical and ephaptic mechanisms. Cones release glutamate in a Ca^2+^-dependent manner, while HCs feed back to cones via an ephaptic mechanism. Hyperpolarization of HCs leads to an increased current through connexin hemichannels located on the tips of HC dendrites invaginating the cone synaptic terminals. Due to the high resistance of the extracellular synaptic space, this current makes the synaptic cleft slightly negative. The result is that the Ca^2+^-channels in the cone presynaptic membrane experience a slightly depolarized membrane potential and therefore more glutamate is released. This ephaptic mechanism forms a very fast and noise free negative feedback pathway. These characteristics are crucial, since the retina has to perform well in demanding conditions such as low light levels. In this mini-review we will discuss the critical components of such an ephaptic mechanism. Furthermore, we will address the question whether such communication appears in other systems as well and indicate some fundamental features to look for when attempting to identify an ephaptic mechanism.

Neuronal activity leads to both intracellular and extracellular potential changes. In this way the synchronized activity of multiple neurons may generate local field potentials (LFPs). LFPs are discussed extensively in the other contributions to the special issue. Here we will discuss a special form of neuronal communication that operates by modulating the extracellular potential: ephaptic communication.

Ephaptic communication differs from LFPs in that it is highly localized and requires a specialized structure, an ephapse. The term ephapse is derived from the Greek verb *ephaptein* which means *to closely touch*. An ephapse is a specialized structure that generates a high extracellular resistance, such that current flowing through the extracellular space produces a potential difference. This potential difference modifies the activity of voltage gated channels localized within the ephapse, leading to, for instance, changes in spike threshold or modulation of synaptic transmission. In principle, any synaptic structure should generate an ephaptic interaction. However, in most synapses the extracellular resistance of the synaptic cleft is not large enough to generate a significant effect (Figure 1A).

**Figure 1:**
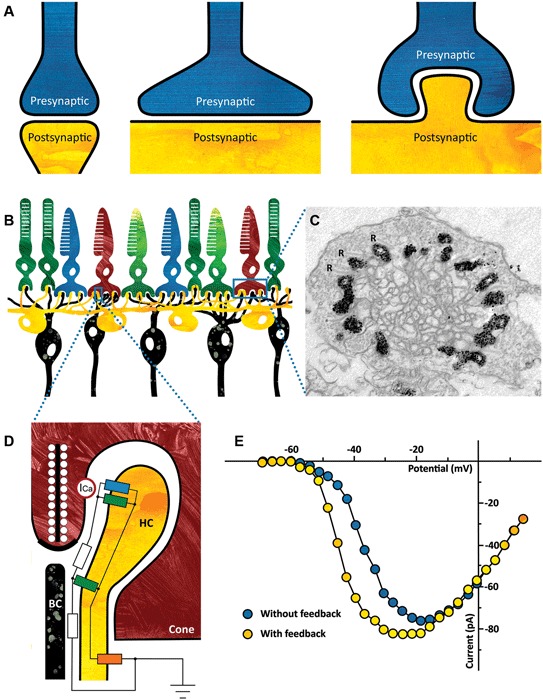
**(A)** Ephaptic interactions will occur in every synapse. The strength of the ephaptic interaction will depend on the organization of the synapse. It will only become significant if the extracellular resistance is high enough. Left: synapse without significant ephaptic interaction. Middle and right: synapse with potentially significant ephaptic interactions. **(B)** Schematic drawing of the vertebrate outer retina. The photoreceptors are the light sensitive neurons in the retina (top layer). They are contacted by horizontal cells (HCs) (yellow) and bipolar cells (BCs) (black). The dendrites of both the HCs and the BCs invaginate the photoreceptor synaptic terminal. **(C)** Electron micrograph of a cone synaptic terminal. In this zebrafish HCs express Green Fluorescent Protein (GFP), which is visible as a black label in this image. Note that every ribbon is flanked by HC dendrites. R: synaptic ribbons. (Taken from: Klaassen et al., 2012) **(D)** Schematic drawing of the cone/HC synapse. In the dark, glutamate release is high and thus the glutamate receptors are activated (green resistors). A constant inward current flows through the connexin hemichannels (blue resistor) and because the resistance of the synaptic cleft is relatively high (white resistors), the synaptic space is slightly negative compared to the extrasynaptic space. When the retina receives a full field light stimulus, cones and consequently HCs hyperpolarize. This causes an increased inward current through the connexin hemichannels, resulting in an increased negativity of the synaptic cleft. The voltage sensitive Ca-channels on the cone (I_Ca_, red circle) detect this as a slight depolarization of the membrane potential, effectively shifting the Ca-current activation potential towards more negative potentials (see Panel E). The influx of calcium increases and consequently the glutamate release. The current entering the HCs via the connexin hemichannels leaves the HCs via the potassium channel on the HC somata (orange resistor). **(E)** Feedback from HCs to cones modulates the Ca-current of cones. Ca-current of a cone in control condition (blue) and when HCs are hyperpolarized and feedback is active (yellow). (Modified from: Verweij et al., 1996).

The retina’s first synapse is a good example of a synapse where significant ephaptic interactions occur (Figure 1B). Cone photoreceptors project to horizontal cells (HCs) and bipolar cells (BCs) via Ca^2+^-dependent glutamate release. Light stimulation hyperpolarizes cones, which leads to a reduction of glutamate release. Since HCs receive input from many cones and are strongly coupled electrically, they collect and average signals over a larger area. This information is fed back to the cones negatively, generating the center-surround organization of BCs. This inhibitory pathway from HCs to cones is mediated via an ephaptic mechanism.

Two key features of this synapse are crucial for the ephaptic interaction to occur. First, a high extracellular resistance is needed, which is generated by a highly specialized synaptic structure (Figure 1C). The dendrites of HCs and BCs invaginate the cone photoreceptor terminal thus creating a restricted synaptic space with a relatively high resistance (Figure 1D, white resistor). Secondly, connexin hemichannels are key players in this ephapse (Figure 1D, blue resistor; Kamermans et al., 2001; Klaassen et al., 2011). Connexins are proteins that can form electrical synapses or gap junctions. However, at the tips of the HC dendrites they form hemichannels, which are non-specific channels that are open at physiological membrane potentials. A constant inward current flows through these connexin hemichannels. This current passes through the synaptic space, which has a finite resistance (Figure 1D, white resistor), inducing a voltage drop and making the synaptic cleft slightly negative. L-type Ca^2+^-channels located in the presynaptic membrane of the cones (Figure 1D, red circle) sense this negativity as a slight depolarization of the cone membrane potential. In a voltage clamp experiment, this will become visible as a shift of the activation potential of the Ca^2+^-current towards more negative potentials. Note that this is an apparent shift, evoked by the change in extracellular potential. Light stimulation hyperpolarizes photoreceptors, leading to a *decrease* in glutamate release, which hyperpolarizes HCs. This hyperpolarization causes the inward current through connexin hemichannels to increase, making the synaptic cleft even more negative. Consequently, the activation potential of the Ca^2+^-current shifts even further, thereby *increasing* glutamate release (Figure 1E, yellow dots).

The ephaptic feedback mechanism has a number of very specific properties. Due to its electrical nature, feedback from HCs to cones is very fast and has no synaptic delay (Vroman et al., submitted). Conventional synaptic transmission depends on vesicular neurotransmitter release. The signal transmitted is noisy because each vesicle release-event causes a discrete postsynaptic potential change. Since ephaptic transmission does not depend on vesicles, it will hardly add noise to the input signal. These features are very well suited for the role of HCs in retinal signal processing. The HC/cone ephapse utilizes ephaptic transmission to generate the surround of BCs, which is the first step in reducing redundant visual information. This redundancy reduction only works if the feedback signal is fast. If this were not the case, the surround of BCs would lag the center response for moving stimuli, thus compromising the efficiency of redundancy reduction. The low noise characteristics of the ephaptic feedback mechanism are especially important at low light levels, when the photoreceptor responses barely exceed the noise level (Field et al., 2005; Ala-Laurila et al., 2011). Adding synaptic noise to the signal would strongly reduce the information content transmitted to BCs.

Are the key features, as we described for the HC/cone ephapse, general requirements for ephaptic communication? A high extracellular resistance is essential but can be achieved in many ways. For instance, it can be achieved by increasing the size of a synapse or by the presence of an invaginating synaptic structure (Figure 1A). However, the extracellular resistance can also be increased by the expression of extracellular matrix molecules such as proteoglycans (Bogdanik et al., 2008; Klaassen et al., 2012). A fundamental property of ephaptic transmission is that it depends on current flow, not on specific channel types. Current will flow through any open channel into the cell, the so-called current sink. While the current sink in the HC/cone ephapse is formed by connexin hemichannels, in other ephapses different channel types may play this role. In our example, L-type Ca^2+^-channels convert the extracellular potential change into a cellular response, but in principle any voltage sensitive channel can play this role. Byzov and co-workers (Byzov et al., 1977; Byzov and Shura-Bura, 1986) were the first to propose an ephaptic interaction between HCs and cones. They suggested that postsynaptic glutamate receptors functioned as current sink. We have shown that, under certain conditions, glutamate receptors can indeed contribute as well (Fahrenfort et al., 2005). In addition, we have shown that pannexin 1 channels also contribute to the ephaptic interaction (Prochnow et al., 2009; Klaassen et al., 2011; Vroman et al., submitted), showing that the ephaptic feedback is mediated by a number of channel types. This large diversity of possible molecular compositions of an ephaptic mechanism might be one of the reasons why so few other ephaptic mechanisms have been described.

Is there evidence for ephaptic interactions in other synapses? For example, the mossy fibers in the hippocampus form large synapses with CA3 pyramidal cell dendrites. These synapses potentially have a high enough resistance to form an ephapse. This ephaptic interaction would depend on the current flowing through glutamate receptors. Activation of presynaptic Ca^2+^-channels leads to glutamate release, which opens glutamate-gated channels in the postsynaptic membrane. The current through these channels makes the potential in the synaptic cleft slightly negative, leading to a depolarization of the presynaptic membrane and a further increase of glutamate release. This positive feedback loop enhances the output of the mossy fiber (Berretta et al., 2000; Kasyanov et al., 2000; Savtchenko, 2007). Interestingly, pannexin 1 channels have also been shown to function postsynaptically from pyramidal neurons (Thompson et al., 2008). Based on morphological arguments, an ephaptic mechanism has also been proposed for synaptic transmission between type I hair cells in the cochlea and the afferent calyx fiber (Hamilton, 1968; Gulley and Bagger-Sjoback, 1979; Yamashita and Ohmori, 1990; Goldberg, 1996). This synapse is also invaginating and thus creates the high resistance necessary for a functional ephapse. Recently, Su et al. (2012) presented evidence for an ephaptic interaction between insect olfactory receptor neurons, located within a sensillium with a high extracellular resistance. They showed that activation of one receptor neuron inhibited the neighboring neuron within the same sensillium, while evidence for synaptic transmission was missing.

An example of ephaptic communication outside the brain can be found in the heart. Action potential propagation between cardiac myocytes, necessary for heart muscle contraction, appears to be dependent on both gap junctional coupling and an ephaptic interaction (Sperelakis, 2002; Lin and Keener, 2013; Rhett et al., 2013). The ephaptic interaction is possible in the area surrounding the gap-junctions, because the space between membranes is narrow and the gap junctions increase the extracellular resistance. Moreover, both connexin hemichannels and sodium channels are present in the membrane to function as current sink. Interestingly, in this system sodium channels not only form the current sink, but also function as the voltage sensitive channels on the opposing cell.

These examples of ephaptic mechanisms are focused on modulation of transmission by extracellular potential differences. However, extracellular potential gradients could have additional influences on synaptic transmission as well. Sylantyev et al. (2008) have shown in hippocampal CA1 cells that the cation influx through postsynaptic alpha-amino-3-hydroxy-5-methyl-4-isoxazoleproprionic acid (AMPA) receptors upon activation by presynaptic glutamate release affect the clearance of glutamate from the synaptic cleft. An increase in AMPA receptor density causes an increase in cation flux. Since glutamate is negatively charged and therefore experiences a force opposite to the cation flux, diffusion from the cleft is faster.

At first instance, it would seem that ephaptic communication is a rather energy intensive mechanism. A continuously open channel puts a high metabolic load on the cell. The cell needs to spend energy to maintain its ionic balance and membrane potential. On the other hand, conventional synaptic transmission is not cheap either. Neurotransmitters have to be synthesized and vesicles have to be generated and filled and require the presence of docking, fusion and recovery mechanisms. On the post-synaptic site, receptors have to be expressed and processes lowering the neurotransmitter content of the synaptic cleft, such as neurotransmitter re-uptake transporters, need to be in place. While an ephaptic system puts a high metabolic load on the cell, it might very well be that conventional synaptic transmission is more costly.

In this mini-review we discussed ephaptic communication within a synapse. We described two main advantages for ephaptic communication: (1) it is very fast and has no synaptic delay, and (2) it has very low noise levels. In principle, such communication will occur in any synapse, but its influence will only become significant if the synapse has certain properties. The elements that are crucial for ephaptic neuronal communication are: (1) an ephapse with a large resistance to the extrasynaptic space, (2) a current sink and (3) voltage sensitive channels on the opposing membrane. Because the identity of the channels involved can be very variable, looking for convoluted or tight synaptic cleft may be a good starting point when searching for an ephapse in other systems.

## Conflict of interest statement

The authors declare that the research was conducted in the absence of any commercial or financial relationships that could be construed as a potential conflict of interest.
